# Supplementary figures and images for: Critical behavioral traits foster peer engagement in Online Mental Health Communities
Source: PLoS One. 2025 Jan 13;20(1):e0316906. doi: 10.1371/journal.pone.0316906 (PMC11729953; doi:10.1371/journal.pone.0316906)

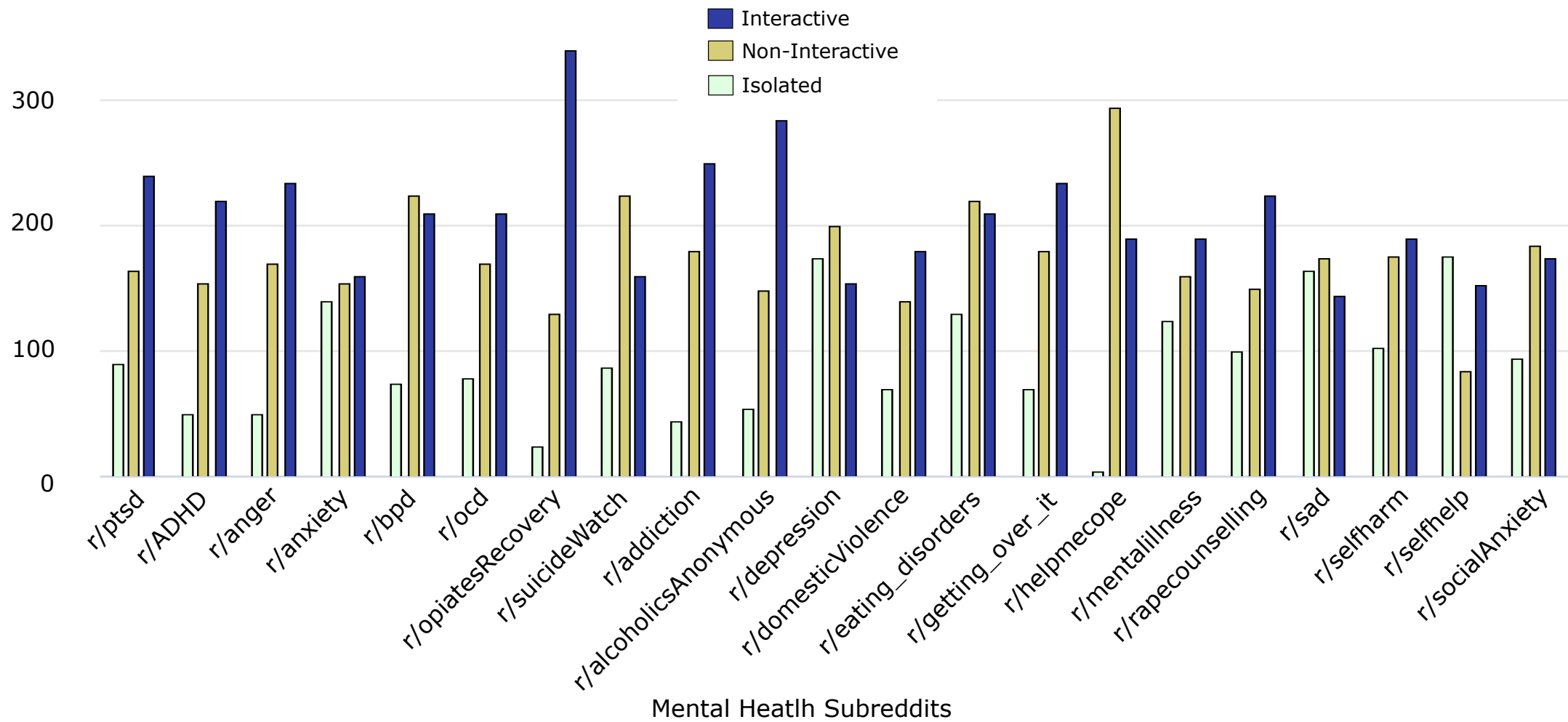

Supplement: S1 Fig — Distribution of mental health subreddits across all engagement categories. (PDF) [file pone.0316906.s001.pdf]
