# Supplementary material for: Critical behavioral traits foster peer engagement in Online Mental Health Communities
Source: PLoS One. 2025 Jan 13;20(1):e0316906. doi: 10.1371/journal.pone.0316906 (PMC11729953; doi:10.1371/journal.pone.0316906)
Supplement: S1 Appendix — Attached is an additional section on a set of auxiliary analysis sections discussing the findings on metadata. (PDF) [file pone.0316906.s002.pdf]

Critical Behavioral Traits Foster Peer Engagement in Online Mental Health Communities (Supplementary Information)

Aseem Srivastava[1,*], Tanya Gupta[1], Alison Cerezo[2,3], Sarah Peregrine (Grin) Lord[2,4], Md Shad Akhtar[1], and Tanmoy Chakraborty[5,6,*]

[1]Department of Computer Science and Engineering, Indraprastha Institute of Information Technology Delhi, New Delhi, India – 110020
[2]Empathy Rocks, Inc. dba mpathic.ai, Bellevue, WA 98004
[3]Department of Counseling, Clinical and School Psychology, University of California Santa Barbara, California 93106, United States
[4]Department of Psychiatry and Behavioral Sciences, University of Washington, 1410 NE Campus Pkwy, Seattle, WA 98195, United States
[5]Department of Electrical Engineering, Indian Institute of Technology Delhi, New Delhi, India – 110016
[6]Yardi School of Artificial Intelligence, Indian Institute of Technology Delhi, New Delhi, India – 110016
[*]Corresponding author, Email: aseems@iiitd.ac.in, tanchak@iitd.ac.in

1 Additional Auxiliary Analysis

This section analyses the auxiliary properties of the BeCOPE dataset. We run fundamental experiments to analyze BeCOPE's focus on metadata and textual properties.

Title Length in BeCOPE

We run a short analysis on the length of the titles of posts from each category in BeCOPE. The aim is to comprehend if more descriptive titles are helpful while receiving help or make it cumbersome for helpers to skip over the post. We find that the mean length of the title for interactive posts is greater than that of non-interactive and isolated posts by a margin of 3.4 and 5.5 words, respectively. These statistics indicate that descriptive titles are more explanatory and likely to receive help. We further conclude that the original poster, who wrote descriptive titles, is likelier to engage with the peers trying to help. This explains the fact that peers primarily look for self-explanatory titles to decide to help.

Body Length in BeCOPE

Once a descriptive title catches the helper's attention, we hypothesize that the body of the text should also be descriptive enough to impart proper information to receive help. To verify this, we run a simple analysis of the body lengths of the post. Furthermore, the statistics show that the mean length of the body for interactive posts (189.95) is greater than that for non-interactive and isolated posts by a margin of 25.64 and 35.24, respectively. This supports our hypothesis, indicating that help-seekers who write descriptive posts are more likely to receive help. We also analyze that the mean body length for non-interactive posts is greater than for isolated posts by a margin of 9.6. Once again, this indicates that posts with longer body lengths are more likely to attract helpers by imparting information better.

The Number of Peer Comments

Here, we analyze how many peers interact with the posts. On average, an interactive post receives 10.12 comments, whereas a non-interactive post receives merely 3.41 comments. Evidently, the number of peer comments on interactive posts is tripled compared to the peer comments on non-interactive posts by a margin of 6.71. This analysis significantly differentiates the interactivity among the interactive and non-interactive posts on mental health subreddits. Moreover, the average number of unique peers interacting on the interactive posts is more than two times the number of non-interactive posts. The same trend holds for other statistical measures. For interactive and non-interactive posts, we observed a standard deviation of 12.47 and 5.94, median of 4 and 2, and 95th percentile of 22 and 10, respectively.

The Number of Unique Peers on a Thread

The engagement category influences the number of peers participating in a conversational thread. Our findings state that an average interactive post attracts 6.07 unique participants on the thread, and a non-interactive post receives merely 3.06. This

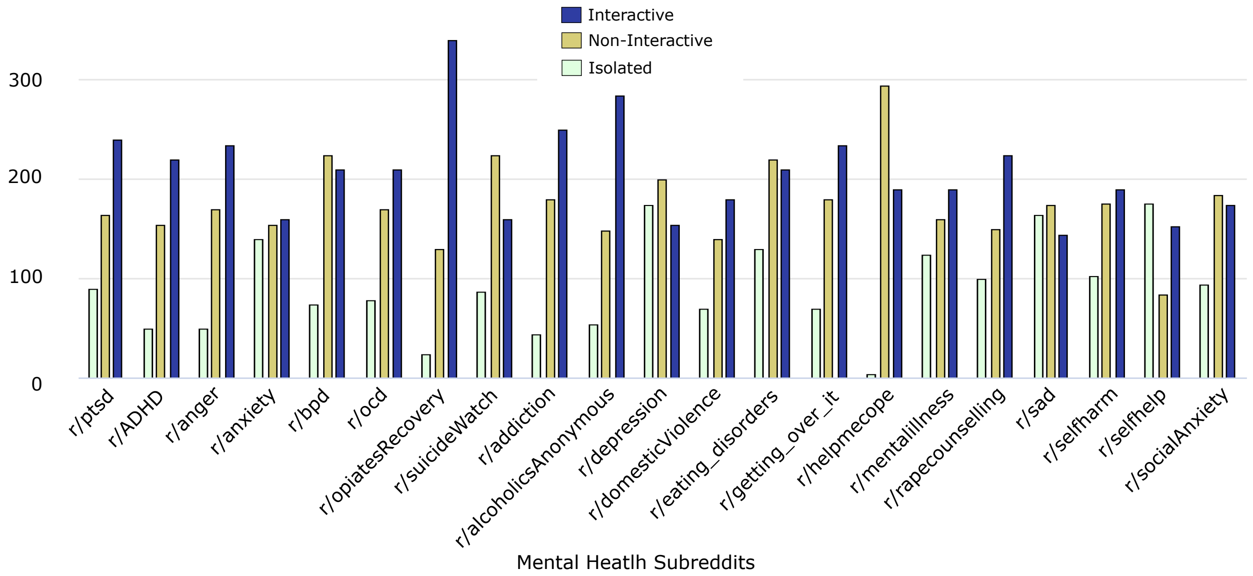

Figure S1. Distribution of mental health subreddits across all engagement categories.

shows that the average number of unique peers on interactive posts is almost double that of peers interacting on non-interactive posts. The help-seeker interacts with the peers by writing replies to their initial replies. The result indicates that the peers are more interactive, and the number of new peers pitching in to help also increased.

To further clarify this, we calculate the standard deviation for the number of unique participants in both categories. For interactive posts, the standard deviation is 2.15, reflecting the variability in peer engagement around the average. In contrast, non-interactive posts had a standard deviation of 1.75, indicating slightly lower variability but still showing a range of engagement levels. Hence, not only do interactive posts relatively attract participants, but the consistency of this engagement is also noteworthy.

Subreddit Division

We assess which mental health issues are more prevalent in peer-to-peer counseling. Also, it is an interesting comparative study to analyze which mental health issues usually attract interaction. As shown in Figure S1, *r/helpmecope* contributes to the minimum number of isolated posts out of all subreddits and the maximum number of non-interactive posts. The subreddit *r/OpiatesRecovery* contributes to the maximum number of interactive posts to the dataset. The number of isolated posts is greater than that of non-interactive posts, which in turn was greater than the number of interactive posts for 13 out of 21 mental health subreddits in the BeCOPE dataset.

Anonymity

Reddit allows users to create *throwaway* accounts, which are temporary accounts for creating just an anonymous post. Thus, any content posted via an anonymous account does not reveal the user's personal account information. To explore the effect of anonymity on peer-to-peer counseling conversations, we extract all the posts with the throwaway user handles from BeCOPE. Next, we analyze these posts for differentiating characteristics. We find 245 posts from throwaway accounts in BeCOPE. Furthermore, 28 out of 245 posts are isolated, 101 are non-interactive, and 116 are interactive. Throwaway accounts contribute to a meagre 2.4% out of total posts. This indicates that help-seekers barely use the added anonymity offered by the OMHC platforms for posting mental health issues. Since it is clear that the posts are less likely to receive a reply if posted from throwaway accounts, we explore the alternate possibility of checking if the help varies with anonymity (Case 1). We also verify if peers' behavior while replying changes when they move from a regular to an anonymous account (Case 2). To this, we test the following two cases:

Case 1: Posts made from throwaway accounts are less likely to receive help as compared to the posts made from any regular account.

Case 2: Help-seekers posting from throwaway accounts are more likely to reply on receiving replies than those posting from regular accounts.

We observe that 88.5% posts by throwaway accounts received a reply compared to 81.3% posts that were not from throwaway accounts. Thus, the finding does not support our hypothesis. Moreover, the original poster replied in 53.5% of

the posts from throwaway accounts, which received at least a reply. This is similar to the 53.8% posts not from throwaway accounts. Consequently, we do not observe any correlation between the behavior of the help seeker and anonymity; thus, the second case also contradicts the findings.

Analysis Based on Similarity

Earlier work showed that better responses from counselors uphold higher linguistic similarity with the clients' responses. Thus, we test the hypothesis to observe if the interactive engagements carry a higher similarity between the post and the comments than other engagements. Hence, to shed light on this and further clarify, we exploit sentence-bert[?] to compute the cosine-similarity between the context-rich BERT embeddings of help-seeker's post and peer comments. As a result, we observe that the earlier work's hypothesis contradicts the findings of our analysis. The average text-similarity between posts and comments for non-interactive posts is very similar to that of interactive posts with a slight difference of 0.0202 only. Thus unlike earlier work, the high textual similarity between the post and the comments is not a standard case.

2 Discussion

The process of data preparation for behavioral analysis in mental health is a critical step in itself. This is due to the multifaceted nature of how individuals express themselves and interact within these contexts. In the BeCOPE dataset, Selecting suitable labels was a careful process that involved the assistance of mental health experts. A team of experts meticulously analyzed a sample of posts, aiming to grasp the overarching posting behaviors demonstrated by peers within mental health-specific subreddits. This analysis was complemented by a thorough review of prior studies in a similar space. Despite the existence of works in understanding OMHC platforms, our work underscores the utmost relevance toward the need for such behavioral analysis while simultaneously presenting a novel perspective compared to the existing works.

Additionally, our study specifically focuses on the mental health context, and the observed effects for intent, readability, and criticism are closely tied to the domain. Reddit's OMHC is a blend of domain-specific discussions and general conversational elements. Our annotation process captures the nuances of mental health interactions, which distinguishes our study from general platform-wide analyses. For instance, readability metric is a platform-wide measure, applicable to general context as well. However, the intent and criticism labels were specifically designed with mental health interactions in mind. The intent categories, such as "seek-help" and "rant," are particularly relevant to mental health discourse, where users often seek support or express against a typical support-seeker's perspective. Similarly, the criticism labels were developed to reflect the nature of feedback in support-seeker's conversations, capturing nuances like self-criticism and criticism directed towards others.

We discussed a sequence of relevant analyses, supplementary to the primary analysis detailed in the the main text. Notably, intrinsic characteristics exhibited by help-seekers significantly influence their engagement dynamics and distinctly bifurcate those receiving interaction from those not. For instance, a recurrent behavioral pattern observed throughout the dataset is the tendency of individuals to post during late hours, presumably when they find spare time from their busy daily schedules. Furthermore, Reddit offers an exclusive feature enabling users to interact incognito (going anonymous while posting). Given the prevailing stigma associated with open discussions in this domain, a prevailing assumption was that engagement in anonymous interactions would surpass non-anonymous posts. Contrarily, our analysis disproved this expectation, revealing that anonymous posts actually attract lower levels of interaction. The crux of our research underscores the need for widespread education regarding the optimal "what, when, and how" of engaging on OMHCs. Such knowledge spread holds the potential to enhance the productive utilization of OMHC platforms and directly impact the efficacy of peer-to-peer support provision among individuals seeking help.
